# Retrospective and prospective perspectives on zoonotic brucellosis

**DOI:** 10.3389/fmicb.2014.00213

**Published:** 2014-05-13

**Authors:** Edgardo Moreno

**Affiliations:** ^1^Programa de Investigación en Enfermedades Tropicales, Escuela de Medicina Veterinaria, Universidad NacionalHeredia, Costa Rica; ^2^Instituto Clodomiro Picado, Facultad de Microbiología, Universidad de Costa RicaSan José, Costa Rica

**Keywords:** brucellosis, *Brucella*, zoonosis, *Brucella*-vaccines, domestication

## Abstract

Members of the genus *Brucella* are pathogenic bacteria exceedingly well adapted to their hosts. The bacterium is transmitted by direct contact within the same host species or accidentally to secondary hosts, such as humans. Human brucellosis is strongly linked to the management of domesticated animals and ingestion of their products. Since the domestication of ungulates and dogs in the Fertile Crescent and Asia in 12000 and 33000 *ya*, respectively, a steady supply of well adapted emergent *Brucella* pathogens causing zoonotic disease has been provided. Likewise, anthropogenic modification of wild life may have also impacted host susceptibility and *Brucella* selection. Domestication and human influence on wild life animals are not neutral phenomena. Consequently, *Brucella* organisms have followed their hosts’ fate and have been selected under conditions that favor high transmission rate. The “arm race” between *Brucella* and their preferred hosts has been driven by genetic adaptation of the bacterium confronted with the evolving immune defenses of the host. Management conditions, such as clustering, selection, culling, and vaccination of *Brucella* preferred hosts have profound influences in the outcome of brucellosis and in the selection of *Brucella* organisms. Countries that have controlled brucellosis systematically used reliable smooth live vaccines, consistent immunization protocols, adequate diagnostic tests, broad vaccination coverage and sustained removal of the infected animals. To ignore and misuse tools and strategies already available for the control of brucellosis may promote the emergence of new *Brucella* variants. The unrestricted use of low-efficacy vaccines may promote a “false sense of security” and works towards selection of *Brucella* with higher virulence and transmission potential.

## INTRODUCTION

**FIGURE 1:**
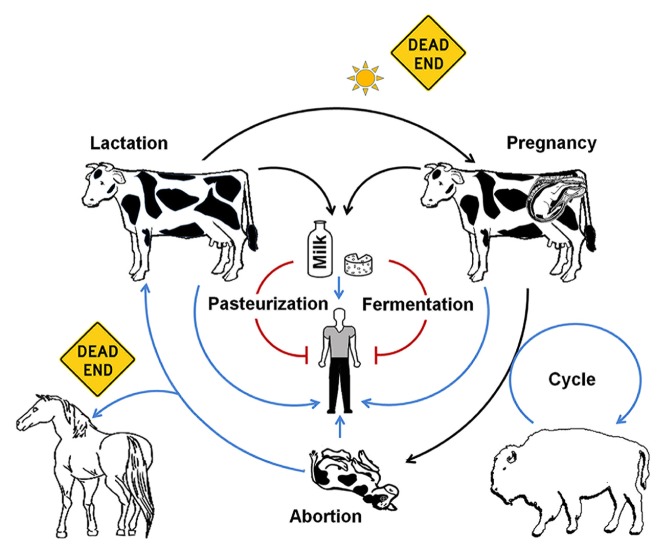
***Brucella* (*B. abortus*) life host cycle.** After host infection the invading *Brucella* replicates within cells of the reticuloendothelial system where it remains for a protracted period of time. After pregnancy, the bacterium invades trophoblasts and the mammary gland. In these sites the bacterium extensively replicates inducing abortion and shedding through milk (black arrows). The heavy contaminated placenta and fetus become the main source of infection for humans and other animal hosts (blue arrows). Humans may acquire the bacterium through ingestion of unpasteurized dairy products. *Brucella* may live up to several weeks, as long as enough organic material is available and the bacterium is protected from the sun’s rays. When exposed to sun’s rays in the open, *Brucella* organisms steadily die (doted black arrow). Pasteurization or fermentation of dairy products eliminates *Brucella* organisms and the risk of human contamination (red blunt arrows). Cross contamination of wild life animals (e.g., bison at lower right) may maintain the bacteria cycling within wild herds, and then of epidemiological relevance. Humans and other animals (e.g., horses) are considered dead ends for the bacterium, and therefore there are not of epidemiological relevance.

Brucellosis is a vicious disease caused by facultative intracellular extracellular pathogens of the genus *Brucella* (Moreno and Moriyón, 2002). The bacterium preferentially replicates within phagocytic cells of the reticuloendothelial system, and in the pregnant animal, inside placental trophoblasts. In domesticated animals, brucellosis is mainly manifested by abortion and epididymitis. Under natural conditions, *Brucella* is horizontally or vertically transmitted. Horizontal transmission occurs through close contact from host to host by means of secretions, sexual intercourse, and more commonly, through liking of aborted fetuses (**Figure 1**). Although *Brucella* has been observed to survive for some time in open environments, the bacterium hardly divides and eventually dies (Crawford et al., 1990). Likewise, some vectors have sporadically been implicated in brucellosis transmission (Gudoshnik, 1958; Dawson et al., 2008; Neglia et al., 2013). However, neither of these two last events plays a significant role in the transmission of brucellosis and they are not of epidemiological relevance (Meyer, 1977; Moreno and Moriyón, 2006).

**FIGURE 2:**
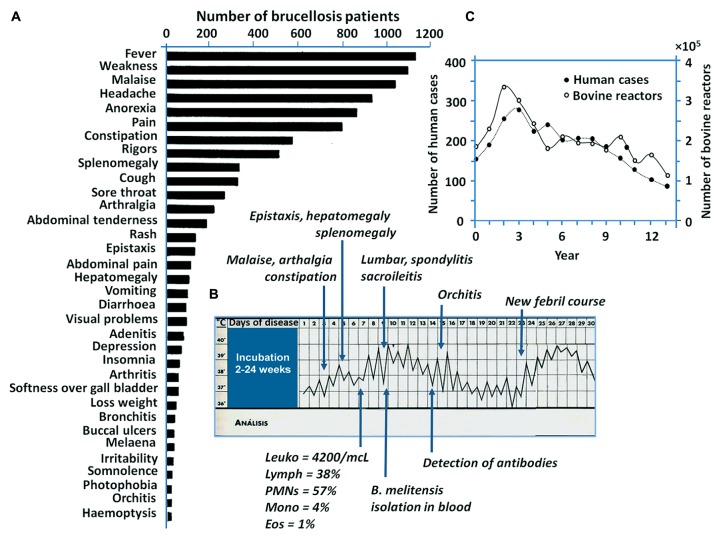
**Brucellosis in humans. (A)** The bar graphic displays the most frequent 34 signs of brucellosis recorded in 1500 patients with proved disease (adapted from Dalrymple-Champneys, 1960). **(B)** The clinical chart displays the typical “undulant fever” suffered by one patient with subsequent clinical signs of brucellosis (adapted from Pedro-Pons et al., 1968). **(C)** Human brucellosis cases and bovines displaying positive *Brucella* infections in United States during 13 year lapse period (1976–1986; adapted from Nicoletti, 1989). In contrast to the silent course of brucellosis in non-pregnant domestic animals, brucellosis in humans courses with a broad collection of clinical symptoms. Notice that the increase and decrease of human brucellosis cases roughly correlates with the increase or decrease of the infection in cattle.

In humans, the disease is more severe than in domestic animals, displaying a collection of clinical symptoms (Dalrymple-Champneys, 1960; Pedro-Pons et al., 1968; **Figure 2**). While there are a few reports of vertical and horizontal transmission between humans (Meltzer et al., 2010; Wyatt, 2010), these are rare events. Therefore, brucellosis in humans is strongly linked to the management of infected animals and ingestion of unpasteurized dairy products (Moreno and Moriyón, 2006; **Figure 1**). In this regard, there is a clear connection of brucellosis with the domestication of even-toed ungulates, milking practices, and fabrication of cheese and other dairy products. It is, therefore, not accidental that lactase persistence – a genetic trait that allows adults to digest lactose from raw milk – has been traced to ungulate domestication places (Sahi, 1994; Enattah et al., 2008; Itan et al., 2010) and in course with the persistence of brucellosis in ancient pastoral people.

At no other time in human history have the changes in technology, domestication and environment been more rapid and so extreme. For thousands of years humans have created new ways of living and social actions have emerged to minimize the effects of infectious diseases. However, domestication and clustering of wild life reservoirs with narrower genetic backgrounds have provided a steady supply of emergent pathogenic organisms. In this regard, brucellosis constitutes an utmost example of a how animal pathogens can emerge as public and veterinary health problems. Here I review how humans have fostered the illness we now call brucellosis that has accompanied civilization since ancient times, when the malady was recognized by its main symptoms: *abortion* and *fever*.

### THE DISCOVERY OF *Brucella* AND BRUCELLOSIS

The seminal discovery of the causative agent of brucellosis, “*Micrococcus melitensis*” (later named *Brucella melitensis*), by the British Surgeon Captain David Bruce, his wife Mary Elizabeth Steele and the Maltese microbiologist doctor Giuseppe Caruana-Scicluna has been eagerly described in many assays (Spink, 1956; Ruiz-Castañeda, 1986; Wyatt, 2000, 2009a). These scientists isolated the bacterium from the liver of diseased soldiers in the Mediterranean island of Malta in 1887, a country that holds prominent megalithic constructions beyond 7000 years old. Following this discovery, the Maltese medical doctor Fioravanti Temistocle Archimede Laurenzo Giuseppe Sammut, better known as “Temi Zammit,” found that the causative agent of Malta fever, Mediterranean fever, Cyprus fever, Neapolitan fever, Gibraltar fever, Crimean fever, Cartagena fever, Rock fever, Barcelonan fever, Corps disease, and undulant fever – just to mention a few names used for this vicious malady – was transmitted from infected goats to humans through contaminated milk (Wyatt, 2005, 2011). Thereafter, Surgeon Captain M. Louis Hughes and Captain James Crawford Kennedy discovered significant details on the zoonotic transmission of brucellosis, including venereal transmission in both humans and animals (Wyatt, 2009b).

Ten years after the isolation of *M. melitensis*, the Danish scientist Bernhard Bag identified “*Bacillus abortus*” (later named *Brucella abortus*) in bovine aborted fetuses (Bang, 1897). Traum (1914) reported the isolation of another organism related to *M. melitensis* (later assigned as *Brucella suis*) from aborted pigs in United States. But the final link of these zoonotic bacteria was accomplished in 1918 by the outstanding American microbiologist Alice Catherine Evans (Evans, 1918). Her achievements helped to understand the epidemiology of brucellosis and contributed to the founding of milk pasteurization as preventive measure. Then, in 1920, Louis Meyer and Wilbur Shaw honored David Bruce and proposed to group these pathogenic bacteria within a single genus named *Brucella* (Meyer and Shaw, 1920).

**FIGURE 3:**
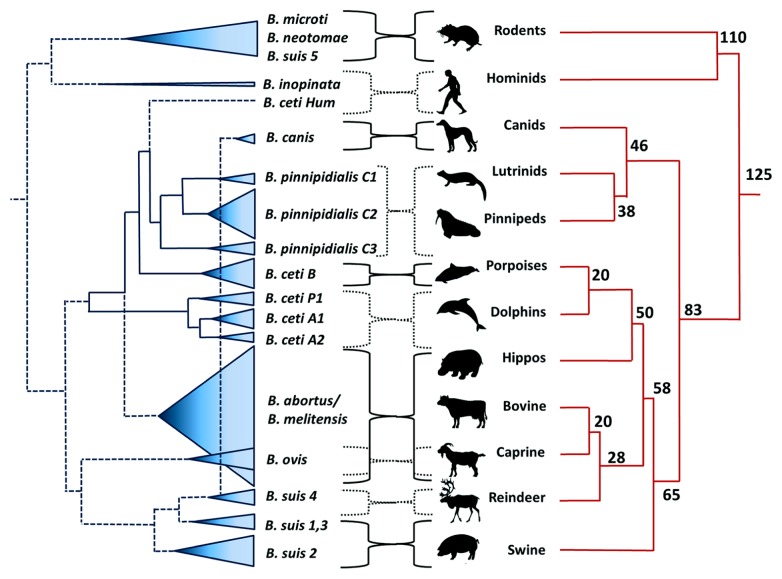
**Dispersion of *Brucella* species confronted to the phylogeny of their preferred host mammal.** The dispersion of the various *Brucella* species is depicted as cones proportional to the number of strains analyzed. The numbers in the mammal phylogenetic tree represent millions of years. *B. suis* biovar 2 also has affinity for hares (lagomorphos). *B. ceti* Hum (human type) does not correspond phylogenetically to *B. ceti* group and this single isolate requires taxonomic definition. The source of the two isolates of *B. inopinata* is unknown. Notice that phylogenetic relationship between the two clades is not perfect suggesting that carnivore mammals acquire brucellosis (probably by depredation) after the initial dispersion of cetaceans and ungulates from an ancestral mesonychid, close to 65–60 million *ya*. Phylogenetic dendrogram was adapted from Guzmán-Verri et al. (2012).

The events that followed all these inspiring investigations have demonstrated the existence of different *Brucella* species (**Figure 3**) that cause brucellosis in domestic animals (cows, sheep, goats, pigs, camels, reindeer, and dogs), wild land animals (bison, elk, hares, muskox, caribou, foxes, and several rodents) and sea mammals (dolphin, whales, seals, and walruses; Godfroid et al., 2011; Guzmán-Verri et al., 2012). Despite of this diversity the only species that are linked to human brucellosis are *B. melitensis*, *B. suis*, *B. abortus*, and to minor extent *Brucella canis* (Moreno and Moriyón, 2006); this last specie being the causative agent of canine brucellosis (Carmichael and Bruner, 1968). Apart from this group there are other *Brucella* strains (e.g., *B. inopinata*) that have been rarely isolated from humans (McDonald et al., 2006; De et al., 2008; Scholz et al., 2010); however, no connection between zoonotic transmission and disease has been established.

Members of the genus *Brucella* are phyllogenetically related to α-Proteobacteria that live in close association with animal and plant cells (Moreno and Moriyón, 2002). From the genotypic perspective the genus is monophyletic with DNA similarity above 97% (Verger et al., 1985). In spite of this, *Brucella* species can be distinguished by single-nucleotide polymorphism analysis, host preference and conspicuous differences in virulence (Bosseray et al., 1982; Foster et al., 2012). In addition, there are several straight forward phenotypic differences, being the most obvious the absence of surface *O*-polysaccharide chain in naturally occurring rough species such as *B. canis* and *Brucella ovis* (Moreno and Moriyón, 2006). One interesting feature of the genus is the absence of plasmids and lysogenic phages, a phenomenon that precludes the horizontal transference of genes through classical routes (Moreno, 1998). Based on this, it has been proposed that the extant *Brucella* species expand clonally within the host environment and that genetic drift depends almost exclusively on mutation and internal genetic rearrangements (Moreno, 1998).

Brucellosis is one of the few diseases in which efficient live bacterial vaccines (e.g., *B. abortus* S19 and *B. melitensis* Rev1) have been developed (Cotton et al., 1933; Elberg and Meyer, 1958). Likewise, through history of microbiology very few diseases have more diagnostic tests than brucellosis (Moreno and Moriyón, 2006). As expected, the isolation of the bacterium stands as the gold standard. However, simple techniques, such as the Rose Bengal test, have survived all challenges and are the most wildly used serological assays (Díaz et al., 2011). This is not by chance, since by the combination of immunization with smooth vaccines, Rose Bengal serological diagnosis and culling of the animals, brucellosis has been controlled and eradicated in many countries of the world (Davidson, 1970; Crawford and Hidalgo, 1977; Whittem, 1978; Wise, 1980; Chamberlin, 1985; Crawford et al., 1990).

## THE EMERGENCE OF ZOONOTIC BRUCELLOSIS

**FIGURE 4:**
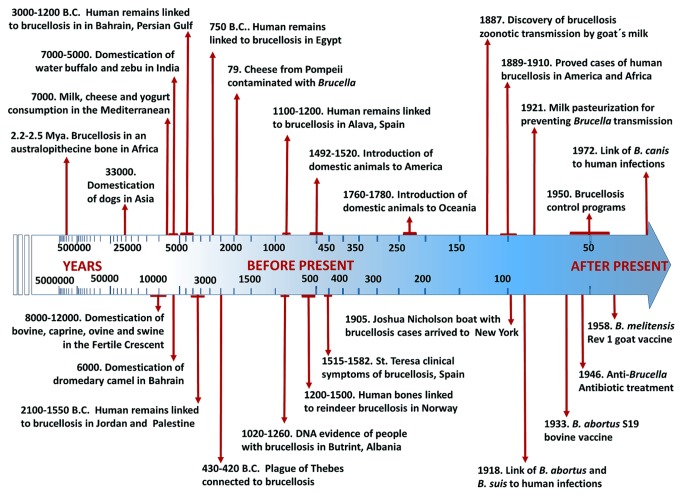
**Timeline of events associated with zoonotic brucellosis.** The scale increases logarithmically from 5 million years in the past to 50 years estimated as the “present” (in 1950). Dates are designated as indicated in the main text.

Through coordinated measures, brucellosis was finally eradicated from the island of Malta 90 years after the discovery of the disease (Wyatt, 2009a). Unfortunately, this has not been the fate of other areas around the Mediterranean Sea, mainly in African, eastern Mediterranean, and Middle East countries, where the disease has been endemic for thousands of years and from which brucellosis was spread around the world (**Figure 4**).

### ZOONOTIC BRUCELLOSIS IN EURASIA AND MIDDLE EAST

Analogous to the island of Malta, Butrint in Albania keeps valuable World Heritage Sites that give testimony on the existence of pastoral inhabitants for millennia (Ryder, 1981). Pathological studies and DNA analysis performed in human remains from graves dated 1260–1020 *ya*, revealed the presence of *Brucella* as the causative agent of the disease that affected these Middle Age inhabitants in the ancient city of Butrint (Mutolo et al., 2012). In addition to Albania, other Balkans countries such as Macedonia and Bosnia and Herzegovina still struggle with animal and human brucellosis; a phenomenon that was boosted by the decline of veterinary and health services in these countries during the political and armed conflicts in the 1990s (Bosilkovski et al., 2010; Puto et al., 2010; Ahmetagic et al., 2012). Human brucellosis outbreaks have also thrived in Balkan neighboring countries such as Greece, Italy, and Turkey (Minas et al., 2007; Mancini et al., 2013). Most likely the disease was endemic in these Mediterranean counties since the beginning of civilization (D’Anastasio et al., 2011). Remains of cheese buried in Pompeii and Herculaneum have been associated with the transmission of brucellosis in Roman imperial times (Capasso, 2002). Likewise, a critical analysis of Thucydides’ history regarding the plague of Athens (2430–2420 *ya*) suggests the presence brucellosis (Kousoulis et al., 2012). Archeological evidence from 7000 *ya* in the eastern Mediterranean region of Anatolia demonstrated ancient skills to transport milk and to manufacture yogurt and cheese, all vehicles for brucellosis contagion (Evershed et al., 2008).

Presumptive human brucellosis cases in skeletal remains from the Bronze Age (4100–3550 *ya*) have been found in Palestine and Jordan (Capasso, 2002; D’Anastasio et al., 2011). It is not coincidental that these places are close to the Fertile Crescent and Taurus Mountains, sites where sheep, goats, cows, and pigs – all known to be preferred *Brucella* hosts – were domesticated between 12000 and 10000 *ya* (Nelson, 1998; Naderi et al., 2008; Pariset et al., 2011; Bonfiglio et al., 2012). Brucellosis has been also implicated in Bronze Age sites located in Bahrain, Persian Gulf (Rashidi et al., 2001; D’Anastasio et al., 2011). This archipelago belongs to a region where the dromedary camel – another common *Brucella* host – was domesticated about 6000 *ya* (Peters, 1997). In this area, human brucellosis acquired through the ingestion of camel dairy products is still endemic, mainly in semi-nomadic Bedouin populations (Rafai, 2002; Shimol et al., 2012). Analyses of human DNA remains from 5000 to 4500 *ya* have revealed that late Neolithic Europeans displayed lower frequency of lactase persistence than modern extant populations (Plantinga et al., 2012). This is compatible with evolutionary pressures related to the consumption of raw milk and consequently with higher chances to become infected with *Brucella.*

Brucellosis is highly prevalent in Asia (Zhang et al., 2010; Denisov et al., 2013; Li et al., 2013). Paleopathological evidence indicates that Lapp people in the Artic area of Northern Eurasia domesticated reindeer (the preferred host for *B. suis* biovar 4) 3000–2000 *ya* or even earlier (Røed et al., 2011; van Kolfschoten et al., 2011) and that these inhabitants also suffered from brucellosis (Ortner, 2003; Røed et al., 2011). Bovine and swine were already present in China, Mongolia, and Korea, at least 5000 *ya* or even before, shortly after their domestication in the Fertile Crescent (Nelson, 1998; Giuffra et al., 2000; Zhang et al., 2013). It seems that water buffalo (*Bubalus* spp.) was also domesticated in China about 4000 *ya* (Teasdale and Bradley, 2012). However, milk and derived dairy products are not commonly found in East Asian cuisines, a culinary activity that is compatible by the lactose intolerance distribution in these populations (Itan et al., 2010). Two exceptions are human groups living in the Asian steppes and Mongolia who still consume milk and fermented dairy products; then keeping lactose tolerance and human brucellosis. It is likely that brucellosis was endemic in these areas before imperial times.

### ZOONOTIC BRUCELLOSIS IN AFRICA AND INDIAN SUBCONTINENT

Human brucellosis is highly prevalent in India (Mantur and Amarnath, 2008). Bovine *Bos indicus* zebu breeds were domesticated in the Indus Valley region (today Pakistan) about 7000 *ya* (Teasdale and Bradley, 2012). An independent domestication of water buffalo was achieved in India about 5000 *ya* (Kumar et al., 2007). Infected water buffalos shed *Brucella* in the milk; however, these animals are more resistant to *Brucella* induced abortion than *Bos* species (Borriello et al., 2006; Adams and Schutta, 2010). An independent domestication of goats also occurred in the Indus Basin in Pakistan already 9000 *ya* (Joshi et al., 2004). Considering these events, it is striking that up to 80% of the Indian population is lactose intolerant. It has been determined that the mutation for lactose tolerance was introduced later on to eastward India from the Middle East (Gallego Romero et al., 2012). This suggests that ingestion of dairy products started later in India than in other regions, and with it, zoonotic brucellosis. Another alternative comes from how Indians prepare their milk: they often ferment it in the form of lassi or paneers, processes that break down the lactose and also kills *Brucella* organisms.

Human brucellosis was described in Mediterranean African countries more than 100 *ya* (Rafai, 2002). It is likely that brucellosis was present in human settlements in Northern Africa already 3000 *ya* and highly prevalent in Egypt during biblical times (e.g., OT, Isaiah 37:8–9, and 2 Kings 19:8–9). Studies performed in Egyptian archeological sites dated 750 B.C. have revealed several human hip bones with signs of brucellosis in this region (Hodgkins, 2002). Brucellosis in southern Africa was detected in dairy herds as early 1913 and the first human cases in 1921 (Bevan, 1931). It seems that the introduction of Indian and Eurasian bovine, sheep, and goat breeds into Africa occurred rapidly after their domestication in the Fertile Crescent. Nevertheless, it has been established that different African ethnic groups have distinct lactase gene mutations that arose independently in different locations between 6800 and 2700 *ya* (Tishkoff et al., 2007). These data fits well with archeological evidence suggesting that pastoral peoples reached eastern Africa in different migration waves, about 4500–3500 *ya*.

It is feasible that brucellosis existed in indigenous African Artiodactyla species (which include a significant number of potential *Brucella* hosts) long before the introduction of domesticated herds. A paleopathological study has suggested the presence of *Brucella* infections in australopithecines, already 2.5–2.3-million *ya* (D’Anastasio et al., 2009, 2011). As expected, this proposal not only has implications on the origin of the disease in local African fauna but, remarkably, also on the feeding habits of these human ancestors. In spite of this, it seems that *Brucella* infections in indigenous African mammals remain low (Gomo et al., 2012) and only relevant when wildlife ungulates become in contact with infected domesticated cows, goats, or sheep (Madsen and Anderson, 1995).

### INTRODUCTION OF ZOONOTIC BRUCELLOSIS IN THE AMERICAN CONTINENT AND OCEANIA

The only indigenous *Brucella* specie in the American Continent seems to be *Brucella neotomae*, first isolated in United States from desert wood rats in 1957 (Stoenner and Lackman, 1957). *B. neotomae* is confined to these rodents with no other known hosts. The absence of domesticated ungulate reservoirs before European colonization very likely circumvented the presence of zoonotic brucellosis in the New World. This is revealed by the close to 100% lactose intolerance of adult Amerindians and in Inuit people (Alzate et al., 1969; Ellestad-Sayed et al., 1978; Sahi, 1994). Thus, it is unlikely that American inhabitants – who populated the continent between 12000 and 4000 *ya* – ingested milk from potential *Brucella* infected wild life ungulates such as bison, muskox, elk, or caribou. Therefore, the various zoonotic *Brucella* species were likely introduced in America during the last decade of the fifteenth century by the first Spaniards conquerors following the arrival of cattle in the colonies (Bowling, 1942). At that time brucellosis was probably highly endemic in the Iberian Peninsula. This is supported by the discovery of human remains from the late Middle Ages displaying pathological signs of brucellosis (Etxeberria, 1994) and by the description of the disease in Spain. For instance the clinical description of the “lousy fever” suffered by the mystic poet St. Teresa of Jesus – born 20 years after Christopher Columbus opened up the Western Hemisphere to European colonization – is compatible with brucellosis (Senra-Valera, 2006).

As for other infectious diseases, the spreading of brucellosis from the “Old World” to the “New World” very likely was a significant outcome of the conquests. It has been well documented that during his second voyage to the American Continent in 1493, Christopher Columbus introduced a significant number of cattle and pigs (de las Casas, 1951). Very probably by these means the introduction of brucellosis in the continent, including the contamination of indigenous fauna such as bison (Rhyan et al., 2013). Brucellosis was detected in a Yellowstone American buffalo herd already in 1917 (Mohler, 1917). Until the first half of the twentieth century, European cows shared with bison herds the same pasture lands (Bowling, 1942) making likely cross infection (**Figure 1**). Indeed, brucellosis in North American bison and elk has been related to cross contamination of bacterial strains (including vaccine strains) from infected European bovine breeds (Meagher and Meyer, 1994; Higgins et al., 2012). Furthermore, the same *B. abortus* biovars (1 and 2) are found in both classes of bovine herds. The disease in the American buffalo is similar to that of domesticated cattle (Rhyan et al., 2001b); though it is believed that bison, like water buffalo, may display some resistance to *Brucella* induced abortion (Herman, 2013).

The origin of *B. suis* biovar 4 infecting Canadian and Alaskan caribou and muskox has been traced to imported reindeer from Siberia, early in the twentieth century (Meyer, 1966; Forbes, 1991). Domesticated reindeer should be also considered a potential source of zoonotic disease since brucellosis – caused by *B. suis* biovar 4 – has been found in Eskimos (Davies and Hanson, 1965; Meyer, 1966; Forbes, 1991). Alternatively, *B. suis* biovar 4 could have arrived with infected caribou and muskox through the Bering Land Bridge during the last glaciation (Campos et al., 2010; Røed et al., 2011).

Human brucellosis was prevalent in Mexico, USA, and Canada for centuries (Spink, 1956; Wise, 1980; Ruiz-Castañeda, 1986). The first human cases in North America were recognized between 1889 and 1894 (Craig, 1903; Gentry and Ferenbaugh, 1911). With the exception of Mexico, nowadays the presence of human brucellosis has become a rare event in northern hemisphere of the American continent. This was the result of the successful pasteurization of dairy products and the application of control programs based in extensive immunization of herds with smooth *Brucella* vaccines, diagnostic tests such as Rose Bengal and complement fixation and efficient culling and management of animal flocks during the second half of the twentieth century (Crawford and Hidalgo, 1977; Wise, 1980). In contrast, the absence of coordinated control programs, poor management of animal flocks, and the introduction of vaccines with low efficacy have kept brucellosis highly prevalent in Mexico, Central America, and most South American countries (Moreno, 2002; Vargas, 2002; Lucero et al., 2008; Herrera-López et al., 2010; Godfroid et al., 2011; Aznar et al., 2012; Román et al., 2013; Rubach et al., 2013).

*Brucella canis* – the last *Brucella* zoonotic specie described – was discovered in Southern United States in the late 1960s (Carmichael and Bruner, 1968). Dogs were the first animals to be domesticated in the world. The earliest archeological vestiges are from Siberia dated 33000 *ya*; while in the American continent the oldest known ancient remains date 11000 *ya* (Leonard et al., 2002; Ovodov et al., 2011). Then, it was expected to find *B. canis* in dog’s wild relatives. However, there are no reports of *B. canis* in wolf or coyote packs and these wild canines seem to display some resistance to smooth *Brucella* species (Davis et al., 1988; Tessaro and Forbes, 2004). Nevertheless, it seems feasible that *B. canis* evolved in dog’s ancestor after predation of *B. suis* biovar 4 infected hosts in Asia (e.g., caribou/reindeer), since these two brucellae species are closely related (**Figure 3**). Moreover, wolves and Artic foxes can become naturally infected with rangiferine brucellosis (Neiland, 1975). As other zoonotic brucellae, *B. canis* might have penetrated to the American Continent during the European colonization. Alternatively, *B. canis* could have traveled in infected dogs through the Bering Strait already 12000 *ya* (Leonard et al., 2002). Presently, canine brucellosis has spread throughout the American Continent (Hollett, 2006; Tuemmers et al., 2013). In any case, the zoonotic potential of *B. canis* is low and just sporadic human cases have been reported in the world (Lucero et al., 2008).

Human and animal brucellosis were very important diseases in New Zealand and Australia as these countries keep large numbers of sheep and bovines. As expected, lactose intolerance occurrence in indigenous people from Oceania is above 95% (Enattah et al., 2008; Itan et al., 2010), a fact that agrees with the absence of indigenous large mammal animals in this region. It is therefore likely that human brucellosis stared with the arrival of infected domestic livestock to Oceania lands in the eighteenth century, through “The First Fleet” and in the ships commanded by Capitan Cook (Gillen et al., 1989). Before this, the only placental mammals (and potential *Brucella* hosts) in Australia were bats, some indigenous rats, mice, and the feral dog named “dingo” introduced from Asia 5000 *ya* (Ardalan et al., 2012). In New Zealand the only placental mammals were bats, *kiore* rats and the Polynesian dog named *kurı^-^*.

Bovine brucellosis was first recorded in New Zealand in 1893 and eradicated 106 years later by an aggressive program that included S19 vaccination, testing, and slaughter of the infected herds (Davidson, 1970). A comparable control program was followed by Australia with a great success (Whittem, 1978; Chamberlin, 1985). The first cases of ram epididymitis caused by *Brucella ovis* were recorded in New Zealand in 1953 (Buddle, 1956). Since *B. ovis* is not pathogenic for humans or other species of animals and mainly affects rams, there are no clear historical records regarding this disease before its discovery. In addition, *Brucella* strains have been isolated in rodents and foxes in Australia (Tiller et al., 2010a; Al Dahouk et al., 2012) and two unconventional *Brucella* strains (one in Australia and one in New Zealand) have been detected in humans (McDonald et al., 2006; Tiller et al., 2010b). In spite of this, no links with the transmission from animals to humans has been established in these cases. Canine brucellosis has just been recently found in domestic dogs in Australia (Gardner and Reichel, 1997; Hofer et al., 2012) but never reported in dingo or *kurı^-^* dogs. Presently, human and animal brucellosis are just sporadic in Australia and New Zealand, remaining feral pigs as the only source of human infections (Eales et al., 2010).

## ARTIFICIAL SELECTION OF *Brucella*

Pathogens and hosts evolve in response to each other and the genetic diversity of both parties represents a pool of possible variants to maintain adaptation via natural selection (Ewald, 2004). Thus, the “arm race” between *Brucella* and preferred hosts has been driven by genetic adaptation of the bacterium virulent systems confronted with the evolving immune defenses of the host. Domestication, anthropogenic modification of wild life and selection of animals by humans are not neutral phenomena. In each event a concomitant selection of the parasitic microbiota occurs (Pearce-Duvet, 2006). Consequently, it is expected that the prevalent extant *Brucella* strains have been selected through “narrow funnels” connected to these processes.

### *Brucella* SELECTION THROUGH DOMESTICATION OF ANIMALS

**FIGURE 5:**
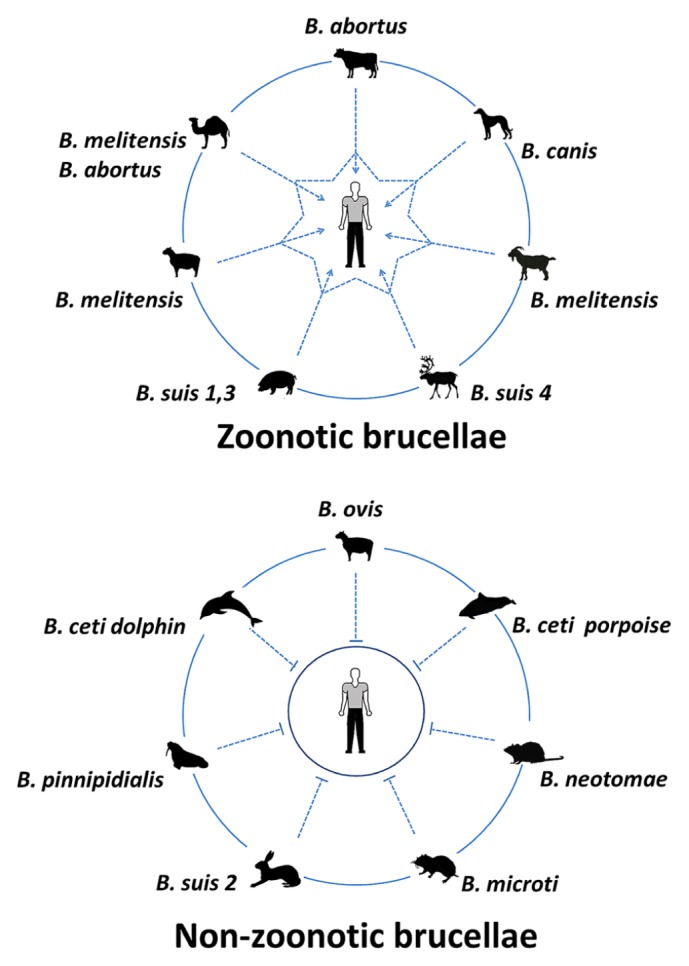
**Zoonotic and non-zoonotic *Brucella* species.** The most virulent species with higher zoonotic spectrum are those from domesticated animals; while those displaying lower pathogenicity and zoonotic potential are those from wild life animals. One exception is *B. ovis* which is a pathogen for rams and does not infect other hosts.

It does not seem by chance that the most virulent *Brucella* species with higher zoonotic spectrum are those from domesticated animals; while those that display lower pathogenicity and zoonotic potential are those from wild life animals (**Figure 5**). Reports of human infections from wildlife reservoirs are scarce. Moreover, within the zoonotic brucellae there are some species that are more virulent than others (e.g., *B. melitensis* > *B. suis* biovars 1, 3, and 4 ≥ *B. abortus* > *B. canis*; Spink, 1956; Bosseray et al., 1982; Ruiz-Castañeda, 1986; Caron et al., 1994). In contrast, *Brucella ceti* and *Brucella pinnipedialis* preferentially infecting free living cetaceans and pinnipeds, respectively, have seldom been found in other animal groups and their zoonotic potential and overall virulence for other animal species, including bovine and swine, seem low (Rhyan et al., 2001a; Perrett et al., 2004; Bingham et al., 2008; Guzmán-Verri et al., 2012). Likewise, *Brucella* species and strains (e.g., *B. neotomae*, *B. microti*, and *B. suis* biovar 5) having preference for wild land mammals are confined to their natural hosts and seldom found in domestic animals or humans (Moreno and Moriyón, 2006; Al Dahouk et al., 2012). Therefore, it is expected that the most prevalent virulent *Brucella* strains were selected during the domestication of animals.

The selection of *Brucella* towards lower or higher virulence has been demonstrated experimentally. Through mutagenesis of genes coding for the so called virulent determinants or regulatory molecules *Brucella* may become attenuated (González et al., 2008; Barrio et al., 2009; Wang et al., 2012). Likewise, by means of genetic manipulation or selection through serial passages into animals, *Brucella* strains can become robust pathogens (Gibby and Gibby, 1965; Jiménez de Bagüés et al., 2010; Grilló et al., 2012; Terwagne et al., 2013).

In addition of displaying host preferences, the various *Brucella* species and strains also form genetic groups that relate with distinctive geographic origins (Le Flèche et al., 2006; Foster et al., 2012; Garofolo et al., 2013; Jiang et al., 2013; Di et al., 2014). This means that *Brucella* clones rapidly expand and transmit within domesticated groups of animals. In spite of their high DNA similarity, the various bacterial species and strains are selected and form discrete family clusters. These observations parallel those showing that some *Brucella* strains may have been removed or minimized from the bacterial pool as consequence of the control programs. Indeed, several *B. abortus* biotypes described decades ago (Crawford et al., 1990; Meyer, 1990) have not been isolated for more than 40 years; instead, predominant variants remain in bovine herds. Therefore, it is feasible that *Brucella* selection towards higher transmissibility and replication occurs through successive infections in confined hosts, as proposed for the evolution of other diseases (Ewald, 2004).

One exception is *B. ovis* (**Figure 5**). Although this bacterium may have been also subjected to selection processes during the domestication of sheep, it remains non-pathogenic for humans or for other animals (Blasco, 1990). In general, rough brucellae such as *B. ovis* are less virulent than their smooth counterparts and have narrower ability to infect other hosts (Moreno and Moriyón, 2006; González et al., 2008). It may be that *B. ovis* was already selected towards a higher affinity for venereal transmission in sheep before domestication of ovine, as suggested before (Moreno, 1992). Moreover, the basal “deep” phylogenetic location of *B. ovis* in relation to *B. abortus*, *B. melitensis*, *B. suis*, and *B. canis* clusters (Foster et al., 2012), also suggests earlier adaptation of *B. ovis* to its host.

### HERD SIZE AND POPULATION DENSITY IN THE SELECTION OF *Brucella*

Other trend that has favored the prevalence and dissemination of brucellosis corresponds to the intensive exploitation of productive animals (Crawford et al., 1990). Humans have taken advantage of the innate social behavior of ungulates and canines and clustered them in small areas. In addition, following anthropocentric purposes, the genetic background of these domestic animals has been narrowed. As in other infectious disease, lower genetic diversity and crowded effect may favor transmission and select for faster replicating organisms with major zoonotic potential (McDaniel et al., 2013). Examples of these were observed in the early days of brucellosis in Malta (Wyatt, 2005, 2009a), and more recently in foodborne outbreaks in Peru (Román et al., 2013) and massive outbreaks in Inner Mongolia, threatening hundreds of thousands of people.

Inner Mongolia, which keeps the largest sheep population (18.2% of the flock), also ranks first in animal and human brucellosis in China (Pu et al., 2009; Mi et al., 2010; Zhang et al., 2010). In 2007, new brucellosis cases were reported in 85 out of 102 districts in Inner Mongolia, with positive prevalence remaining in the other 47 districts. From 1996 to 2010, 78246 human cases were detected with 90% of the new cases reported between 2005 and 2010. This accounts for 40% of the near 200000 cases detected in China for this period. In 2010, this figure reached 47.2%. According to various models, this may be just the “tip of the iceberg” and it is expected that the number of human cases will increase dramatically in the following years (Hou et al., 2013). Moreover, it has been demonstrated that in endemic areas about 20% of the infected individuals remain undiagnosed. Indeed, family members of the patients with brucellosis are under increased risk of acquiring the disease (Tabak et al., 2008). Thus, family screening in endemic areas is recommended.

Novel circumstances for fast transmission of zoonotic brucellosis have also been observed in confined semi-nomadic Bedouins infected from camel’s milk (Shimol et al., 2012; Shemesh and Yagupsky, 2013) and commercial dog kennels. Camels cohabiting with goats and sheep in small areas are becoming a common practice in Middle East and Arab countries. Dog packs seldom exceed more than a dozen individuals. Consequently, in crowded kennels *B. canis* spreads rapidly inducing massive abortions in bitches, testicular degeneration in males, and becomes a zoonotic risk (Lucero et al., 2008; Gyuranecz et al., 2011; Reynes et al., 2012; Marzetti et al., 2013). Therefore, intensive exploitation and clustering of animals in poor epidemiological control conditions may favor selection for faster *Brucella* transmission and zoonotic disease.

### SELECTION OF *Brucella* IN WILDLIFE ANIMALS

Distinct *Brucella* clusters have also been identified in wild life animal populations located in areas separated by natural barriers (Forbes, 1991; Maquart et al., 2009; Guzmán-Verri et al., 2012). As with domesticated species, anthropogenic modification of wild life may also have narrowed the genetic diversity, impact host susceptibility and pathogen transmission. A noteworthy event has been the threatening of the American buffalo which was close to extinction (Hornaday, 1889). Thus, the prevailing bison herds have been founded by a small group of few surviving individuals (Gross and Wang, 2005). This is relevant since North American bison herds remain infected with *B. abortus* (Rhyan et al., 2001b). The European counterpart of this incident corresponds to the Alpine ibex (*Capra ibex*). Historically these wild goats were endemic throughout the European Alps. Due to excessive hunting and constrain of their natural habitat, the ibex herds in Central Europe declined to low dangerous numbers. As consequence, the founding of new ibex herds in the Alps come from a pool of few animals, narrowing their genetic diversity (Biebach and Keller, 2009). In certain areas ibex herds are infected with *B. melitensis* strains displaying also high seroprevalence (Ferroglio et al., 1998; Mick et al., 2014). Therefore, these wild goats may become a source for the reintroduction of *B. melitensis* in domestic ruminants and humans in Central Europe (Mailles et al., 2012; Hars et al., 2013; Rautureau et al., 2013).

Another example relates to the hunting of marine mammals, linked to the overexploitation of their natural food resources and contamination of the seas. These negative activities have promoted clustering of different *Brucella* infected marine mammals in reduced areas where food is available, causing excessive competition, undernutrition, stress, and immunosuppression (Ohishi et al., 2008; Van Bressem et al., 2009). As revealed by the increasing brucellosis case reports in some species of cetaceans over others (Maquart et al., 2009; Guzmán-Verri et al., 2012), these unnatural conditions may favor the selection of *Brucella* organisms with higher transmission rate.

*Brucella* divergence seems linked to selective forces within the host environment, and consequently, to the evolution of the host (Moreno, 1998). However, this constrain is not absolute and *Brucella* species living in wild life or in semi-domesticated hosts may still qualify as potentially pathogens for humans and domestic animals (Godfroid et al., 2011). The phenotypes of *B. ceti*, *B. pinnipedialis*, *B. microti*, and *B. neotomae* correspond to smooth types equipped with all known “virulent” factors (Audic et al., 2009; Guzmán-Verri et al., 2012). Up to now “mysterious” subtle differences with the classical zoonotic *Brucella* have kept these other wildlife species out from causing disease in humans. But the correlation of the various species in relation to host preference is not perfect and phylogenic patters suggest that *Brucella* organisms are capable to breakdown the species barrier and “jump” from one mammal order to a very differ one (**Figure 3**). Eventually, this might favor the persistence of a distinct *Brucella* clone in a different “preferred” host.

## COPING WITH BRUCELLOSIS

In the presence of brucellosis, management becomes highly demanding (Spink, 1956; Ruiz-Castañeda, 1986; Moreno and Moriyón, 2006). Domesticated animals and humans have coexisted for millennia without significant intervention measures to control the disease. It is likely that a large part of the prevalent *Brucella* zoonotic species was selected in flocks during this long-lasting initial period. In some regions of the world, mainly in low income countries, these weak control actions are still common (Rubach et al., 2013). It is likely that a fraction of the genetic background of both humans and animals has been also shaped during the coexistence with *Brucella* organisms; mainly nearby to the regions where domestication took place (Pashaei et al., 2009; Asaei et al., 2013; Rasouli et al., 2013).

### ERADICATING BRUCELLOSIS

After the discovery of *Brucella* organisms and their mode of transmission, direct measures toward the control and eradication of the disease were taken in several countries. As stated, killing of the bacterium by milk heating was one of the first procedures that prevented the transmission of brucellosis. A second relevant action was the discovery of diagnostic techniques capable to distinguish infected animals (Alton et al., 1988). Third, was the development of efficient vaccines for protecting bovine, caprine, and ovine herds (Cotton et al., 1933; Elberg and Meyer, 1958). In addition, in some areas systematic slaughtering of the infected animals reduced the density of the bacterium (Ebel et al., 2008). Though, the control of brucellosis by the sole action of culling the infected animals is extremely expensive and not practical under high disease prevalence conditions (Moreno, 2002; Office International des Épizooties, 2013). Following this, massive vaccination in combination with serological diagnoses and culling of the infected animals has become the chief strategy for the control of brucellosis (Office International des Épizooties, 2013). Countries where brucellosis has effectively been controlled have used the following procedures: reliable live vaccines (e.g., S19 and Rev1), adequate immunization protocols (e.g., single dose vaccination, reduced dose), extensive protection coverage (e.g., 100% of the herds at risk), suitable diagnostic tests (e.g., Rose Bengal, RID, Complement fixation, iELISA), sustained removal of the infected animals and restriction in the traffic of animals from infected herds to free herds (e.g., control transhumance herds; Davidson, 1970; Whittem, 1978; Wise, 1980; Moreno and Moriyón, 2006; Ebel et al., 2008). Accordingly, these countries have also narrowed the genetic pool of virulent brucellae and succeeded in eradicating human brucellosis.

### THE BASIC REPRODUCTIVE NUMBER AND SELECTION OF VIRULENCE THROUGH VACCINATION

**FIGURE 6:**
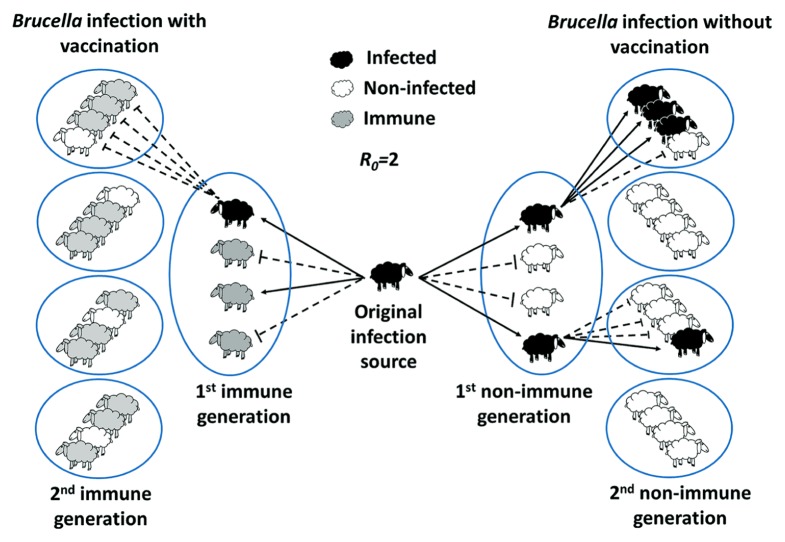
**Herd immunity theory and the basic reproductive ratio (*R*_0_) in *Brucella* herd infections.** Herd immunity theory proposes that the protective effect of *Brucella* vaccinated individuals in a given population extends beyond to unvaccinated population. *R*_0_ corresponds to the average number of new *Brucella* infections caused by single infected source. If acquired immunity is present in the herd, the population is no longer entirely susceptible. The greater the proportion of individuals is immune to *Brucella*, the smaller the probability that a susceptible host will come into contact with an infectious animal. Then, the transmission from one animal to other is likely to be disrupted when an appropriate number of the population (predicted on the basis of *R*_0_) are immune to the bacterium. For instance, if *R*_0_ = 2 (an estimated *R*_0_ for *B. melitensis* transmission in sheep), then a geometric increase in infections occurs over time (right panel). If 75% of the population is protected by the vaccine (minimal protection rate estimated for Rev1 vaccine), then the bacteria fails to grow in the host animal and be transmitted (left panel). It is predicted that vaccines with lower protection rate require larger coverture and greater actions of culling of the animals. New productive infections are depicted by black solid arrows; unproductive transmission is indicated by dashed blunt arrows.

The basic reproductive number, also known as *R*_0_, is the average number of secondary infections arising from one infected individual in a completely susceptible animal population (Gandon et al., 2001). That is, for the disease to spread and for an effective animal to animal *Brucella* transmission it is required that the pathogen’s *R*_0_ exceeds 1 (**Figure 6**). In contrast if *R*_0_ < 1, then the disease has the tendency to fade away. Higher the *R*_0_ value, higher will be the number of subsequently infected individuals. Concomitantly, larger and denser the population of susceptible individuals higher would be the chances for the pathogen to achieve a steady and successful adaptation in the host. As consequence of a collection of unsuccessful events in many middle and low income countries (Moreno, 2002; Blasco and Moriyón, 2005), the *R*_0_ value exceeds 1; thus keeping the disease and the zoonotic potential high (Vargas, 2002; Godfroid et al., 2011; Aznar et al., 2012; Chand and Chhabra, 2013; Denisov et al., 2013; Jiang et al., 2013; Li et al., 2013; Rubach et al., 2013).

Yet, brucellosis is a complex disease and significant political and economic interests are often in play (Moreno, 2002; Pappas and Memish, 2007; Lundquist, 2012). Of all the problems in control programs, the introduction of low protection rate vaccines stands as a major drawback (Blasco et al., 1993; Verger et al., 1995; Moriyón et al., 2004; Godfroid et al., 2011). Apart from their failure in controlling brucellosis, there are long-term consequences in the use vaccines with low efficacy. In this direction a variety of evolutionary scenarios are possible (Gandon et al., 2001; Gandon and Day, 2007), including the selection of more virulent *Brucella* strains.

Effective vaccination limits *Brucella* infection, restricts shedding, hampers transmission from animal to animal and diminishes the risk of zoonosis (Nicoletti, 1990). In addition, when combined with removal of positive infected animals, efficient vaccination may select for breeds with higher resistance against the disease (Adams and Schutta, 2010). Immunization with efficient vaccines may replace natural infections by inducing competent immunity (Plommet et al., 1987); likewise, culling of the infected animals replaces the natural selection of hosts displaying reproduction impartments, such abortion, placenta retention, and infertility (Fogel and Fogel, 2011). Eventually, these sustained combined strategies establish a *R*_0_ < 1 with the concomitant peter out of the disease. Moreover, when *R*_0_ < 1 the pathogen evolution rate towards higher virulence may be overcome and virulent field *Brucella* strains eradicated from domestic flocks (Davidson, 1970; Whittem, 1978; Wise, 1980; Moreno and Moriyón, 2006; Office International des Épizooties, 2013).

**FIGURE 7:**
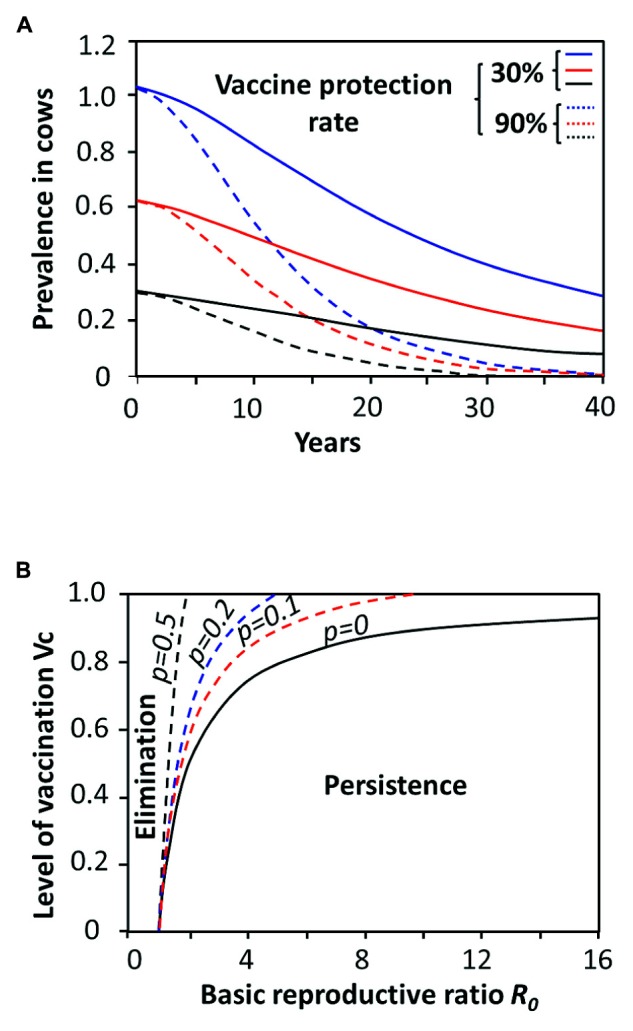
**Sceneries for vaccine performances against brucellosis according to various models. (A)** Predicted model for bovine brucellosis eradication in Mato Grosso (blue), Rodôni (red), and Goiás (black) Brazilian States with different experimental prevalences using two vaccine protection rates. Protection rate by low-efficacy vaccines or low coverage vaccination are not capable to eradicate brucellosis in four decades (solid lines), independently of the initial prevalence. The critical threshold applies to both: (i) the proportion of the population that needs to be vaccinated, and; (ii) the protective quality of the vaccine (adapted from Amaku et al., 2009). **(B)** Prediction for the elimination or persistence or of brucellosis according to *R*_0_ and the critical level of vaccination *V*_c_. The *V*_c_ needed to protect a given population of animals is calculated by *V*_c_ = 1 - 1/*R*_0_. Those vaccines that fail to generate immunity in a fraction *p* of the immunized individuals, require higher coverage defined by *R*_0_ - 1/*R*_0_(1 - *p*). However, if *p* is too big it may be impossible to eradicate the *Brucella* infection. Parameters such as culling of the infected animals and diminishing of the density of the susceptible animals have a significant impact in both **(A)** and **(B)** since by reducing the value of *p* (not shown). The solid black line represents the outcome of an ideal no “leaking” vaccine (adapted from Keeling et al., 2013).

In contrast, inefficient vaccines currently used in many countries for the control of bovine, sheep, or caprine brucellosis might work in the opposite direction. Indeed, the protection afforded to non-immune animals by the presence of sufficient numbers of immune individuals, known as “herd immunity” (**Figure 6**) is threatened if the immune status of the herd is low. That is, inefficient vaccines may promote a fertile niche in weakly immune hosts allowing virulent *Brucella* to be transmitted though vaccinated animals (Herrera-López et al., 2010, 2011; Arellano-Reynoso et al., 2013). In curse this will increase the number of secondary infections. For example, if the anti-*Brucella* vaccine fails to generate immunity in a fraction *p* of those animals vaccinated, then to achieve herd immunity we need to vaccinate a proportion of individuals equivalent to *R*_0_ - 1/*R*_0_(1 - *p*) (**Figure 7**). Hence, if *p* is too big it may be impossible to eradicate brucellosis as it has been the case in many countries where vaccines of low-efficacy have extensively been used (Blasco et al., 1993; Moreno, 2002; Vargas, 2002; Blasco and Moriyón, 2005; Arellano-Reynoso et al., 2013; Chand and Chhabra, 2013; Denisov et al., 2013; Hou et al., 2013; Jiang et al., 2013; Li et al., 2013; Oseguera-Montiel et al., 2013; Rubach et al., 2013).

**FIGURE 8:**
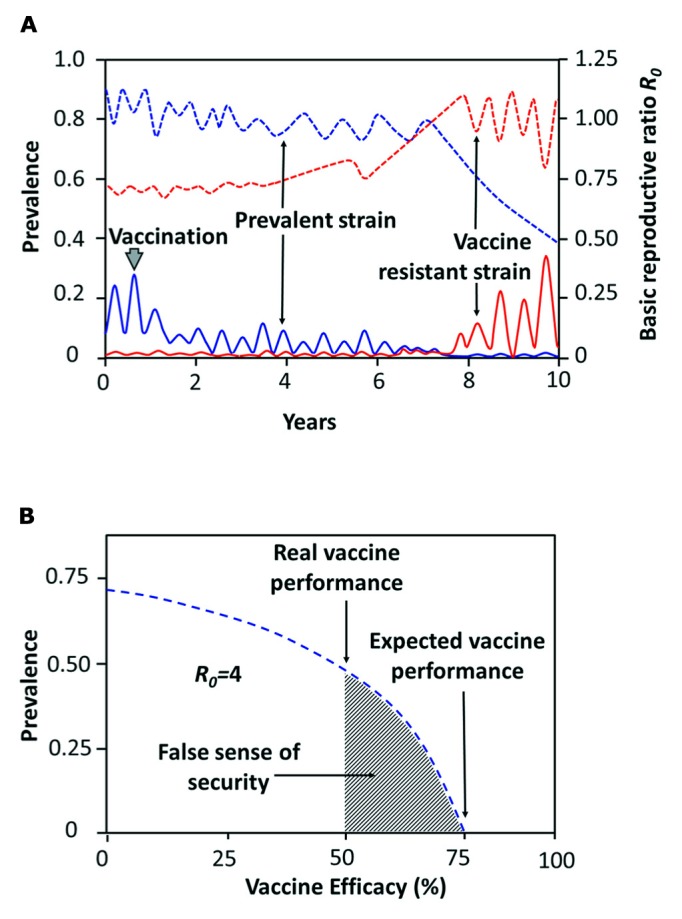
**Prediction for emergence of resistant-vaccine *Brucella* strains and the false sense of security. (A)** Immunization with low efficacy vaccines may change the competitive balance between *Brucella* virulent strains. Before vaccination (6–8 months of age) one prevalent strain is observed (blue line). After vaccination of 100% of the susceptible animals with a low efficacy vaccine that only gives 30–40% protection rate, the vaccine-resistant strain (red line) may eventually emerge with a competitive advantage that is only evident after a large proportion of the population has been vaccinated over the years. The vaccine resistant-strain arises from the *Brucella* pool, either through mutation of the prevalent strain or by selection of previously existing strains. Only after the *R*_0_ of the vaccine-resistant strain has exceeded that of the prevalent strain, then a new brucellosis epidemic event develops. Solid blue and red lines correspond to the prevalence ordinate; dashed lines correspond to the *R*_0_ ordinate (adapted from Scherer and McLean, 2002). **(B)** Reduction in brucellosis prevalence below the critical vaccination threshold (expected vaccine performance) with an anti-*Brucella* vaccine efficacy of 75% and *R*_0_ = 4. In a bovine close homogeneous population a lower value for *R*_0_ would be associated with a lower *Brucella* prevalence. The false sense of security (shadow area) for a given vaccine lays between the expected vaccine performance (e.g., 75%) and the real vaccine performance (e.g., 50%).

In cases in which the relative fitness of competing pathogens depends on the immune status of their host, low-efficacy vaccines inducing responses below the protective threshold may also prompt pathogen evolution towards higher virulence (**Figure 8**; Read and Mackinnon, 2008). Selection pressures may work in the same direction observed for non-sterilizing antibiotic treatments, in which the surviving microbes may display a higher resistance edge (Davies and Davies, 2010). Furthermore, anti-*Brucella* vaccines lacking some fundamental virulent molecular determinants or displaying a large collection of mutations (Wang et al., 2012), give a competitive advantage to virulent strains possessing full set of these factors, as it has been already shown for rough *Brucella* strains devoid of *O*-polysaccharide antigen (González et al., 2008; Barrio et al., 2009; Herrera-López et al., 2010).

### ANTI-*Brucella* VACCINES AND A FALSE SENSE OF SECURITY

In certain contexts vaccination induces a “sense of security” in non-specialized general public. This sense of security is sustained in the trust and faith that people have developed on vaccines that successfully prevented and eradicated diseases. If the vaccine is highly efficient, then the faith and trust is justified and not harm is done. However, this complacency is particularly dangerous when vaccines with low efficacy and short-term protective duration are introduced; then, a “false sense of security” may be generated, mainly when the information is not given properly (Henderson et al., 2011). Generally speaking, the false sense of security lays between the optimal expected efficacy for a given vaccine and the real performance of that vaccine (**Figure 8**) and it has a direct impact in the assessment of herd immunity. The use of anti-*Brucella* vaccines displaying low efficacy could generate a false sense of security in the minds of livestock farmers and Veterinary Health authorities, who may believe that herds are fully protected.

Under low threshold immunity conditions the host becomes a favorable environment for the replication and spread of field bacterial strains (Moreno, 2002; Herrera-López et al., 2010, 2011; Arellano-Reynoso et al., 2013; Denisov et al., 2013; Jiang et al., 2013) and a potential niche for *Brucella* selection. This is particularly relevant when prevalence is high and surveillance is low to begin with and when the favored virulent microbe emerges within a restricted population. These arguments are supported by several mathematical and epidemiological models (Gandon et al., 2001; Scherer and McLean, 2002; Day and Gandon, 2007; Gandon and Day, 2007).

### ANTIBIOTICS AND *Brucella*

In the light of unrestricted use of antibiotics the emergence of antibiotic resistant *Brucella* clones should not be excluded *a priori*. However, in contrast to other bacterial pathogens, antibiotics do not seem to play a significant selective role in brucellosis. Due to economical, epidemiological, and public health reasons, treatment with antibiotics has been precluded in productive animals with brucellosis (Guerra and Nicoletti, 1986; Radwan et al., 1993; Office International des Épizooties, 2013). One exception is canine brucellosis. Pets with brucellosis are frequently treated with antibiotics, not always with success (Ledbetter et al., 2009). Antibiotics have also been used in brucellosis research for selecting specific strains displaying antibiotic resistance (Schurig et al., 1991; Adone et al., 2005; Ravanel et al., 2009), This has also important implications in the accidental transmission of *Brucella* organisms in the laboratory and the potential role of this bacterium as biological weapon (Yagupsky and Baron, 2005). In spite of this, most *Brucella* clinical isolates remain susceptible to the classical antibiotics used for treatment of brucellosis (Guerra and Nicoletti, 1986; Ayaşlıoğlu et al., 2008; Maves et al., 2011; Abdel-Maksoud et al., 2012). In broad terms, people constitute a dead end for *Brucella* transmission (Spink, 1956; Ruiz-Castañeda, 1986); therefore, antibiotic treatment of infected humans is not epidemiologically relevant (**Figure 1**). This phenomenon may relate to the absence of plasmids and lysogenic phages in *Brucella* organisms, as it has been explained elsewhere (Moreno, 1998). In this sense, the short-term emergence of antibiotic resistant *Brucella* does seem plausible.

## CONCLUDING REMARKS

The capabilities of *Brucella* to infect and propagate in the preferred hosts follow at least five stages: (i) ability to invade; (ii) power to circumvent the initial defenses; (iii) competence to replicate; (iv) capacity to be transmitted; and (v) endurance to be maintained within the host population (Moreno and Moriyón, 2006; Martirosyan et al., 2011). How, when, and where pathogens cross the boundaries that separate their natural hosts from human populations and provoke an epidemic disease, is not entirely known. Human-to-human *Brucella* transmission would require that the pathogen’s *R*_0_ exceeds 1 (Gandon et al., 2001). Although *Brucella* animal pathogens have already achieved the first three stages in humans and in occasions the fourth stage (Meltzer et al., 2010), still the disease in humans is terminal and human mediated transmission is not of epidemiological importance (**Figure 1**). Thus, *Brucella* has not yet reached the *R*_0_ threshold to emerge as permanent pathogen within human populations and contagion remains dependent on animal reservoirs. However, under these circumstances human brucellosis may display a *R*_0_ above the threshold that depends on the zoonotic infection rate. For instance, as consequence of high prevalence in domestic animal reservoirs (sheep) in Inner Mongolia, the *R*_0_ for human infection corresponds to 1.8 (Hou et al., 2013). Under the prevailing control measures and use of low protective vaccines (Blasco et al., 1993; Verger et al., 1995) it was predicted that human brucellosis will continue to increase for the next decade in China.

Ecological factors and human activities may influence and induce changes in the microbial virulence patterns. But to distinguish *Brucella* clones displaying higher virulence is not an easy task (Moreno and Moriyón, 2002). *Brucella* organisms lack classical molecular markers commonly used to trace virulence such as toxins, fimbria, plasmids, capsules, antigenic variation or resistant forms. The so called “virulent factors” are intertwined with the overall *Brucella* structure and physiology (Moreno and Moriyón, 2002; Barbier et al., 2011) and are found in practically all *Brucella* species examined, independently of their pathogenicity for humans (Audic et al., 2009, 2011). Moreover, many of the molecular determinants such as cell envelope components, secretion systems, regulatory systems, transporters, and effectors assigned as virulent factors are also found in soil bacteria related to brucellae (Barquero-Calvo et al., 2009). As stated before, *Brucella* species form a compact genetic cluster and display host preference commensurate with their phylogenetic dispersion (Maquart et al., 2009; Audic et al., 2011; Foster et al., 2012). Therefore, the major scientific challenges that brucellosis research confronts relate to the identification of those discrete genotypic and phenotypic changes that have favored the adaptation to the preferred hosts and those molecular determinants that have made some *Brucella* species more virulent than others. In addition, efficient vaccines for dogs, pigs, water buffalo, and camels, as well as for some wild life animals, are required (Godfroid et al., 2011).

During the first half of the twentieth century through the early 1980s, efficient live *Brucella* S19 and Rev 1 vaccines for preventing brucellosis were developed together with robust procedures for testing their safety and efficacy (Cotton et al., 1933; Elberg and Meyer, 1958; Crawford and Hidalgo, 1977; Alton et al., 1988; Moreno and Moriyón, 2006). In addition, various inexpensive and straightforward serological tests as well as good management strategies were successfully implemented (Crawford and Hidalgo, 1977; Alton et al., 1988). Those were the days when brucellosis control programs succeeded in many parts of the world (Crawford and Hidalgo, 1977; Plommet, 1992). Circumstances have changed and the global agenda has been modified towards other interests. Taking into account that ignorance persists and economic profits pursue without other considerations, it is difficult to envision what will happen and how biological and cultural evolution will shape brucellosis and human battlement against this zoonotic disease.

For much of the twentieth century the misuse of antibiotics has taken place with little concern on the evolutionary consequences and selection of antibiotic resistance hypervirulent bacterial strains (Davies and Davies, 2010). It is our contention that we should not repeat that complacency with misuse of poor brucellosis vaccines, dubious immunization protocols, expensive diagnostic tests, and inadequate management procedures.

## AUTHOR CONTRIBUTIONS

Edgardo Moreno wrote and revised the manuscript and made the figures.

## Conflict of Interest Statement

The author declares that the research was conducted in the absence of any commercial or financial relationships that could be construed as a potential conflict of interest.
